# Supplementary material for: Fatty Acid β-Oxidation May Be Associated with the Erythropoietin Resistance Index in Stable Patients Undergoing Haemodialysis
Source: Diagnostics (Basel). 2024 Oct 16;14(20):2295. doi: 10.3390/diagnostics14202295 (PMC11506985; doi:10.3390/diagnostics14202295)
Supplement: Supplementary file 1 [file diagnostics-14-02295-s001.zip › diagnostics-3232088-supplementary.pdf]

Supplementary materials

Fatty acid β-oxidation may be associated with the erythropoietin resistance index in stable haemodialysis patients

Patients and Methods

The study recruited 167 patients receiving maintenance HD treatment at Fujita Memorial Hospital. The exclusion criteria were as follows: (1) no treatment with recombinant human erythropoietin, (2) treatment with L-carnitine, (3) active infectious diseases, (4) active liver disease or cancer, (5) recent blood transfusion or surgical procedure, and (6) active haemorrhage. As a result of these exclusions, the full range of Hb and free carnitine values in the study participants were 8.0 to 13.1 g/dL (19 patients with Hb levels above 12.0 g/dL) and 7.39 to 54.71 nmol/mL (20 patients with free carnitine levels of 36 nmol/mL or more).

Based on the Guidelines for Renal Anemia in Chronic Kidney Disease (2008 Japanese Society for Dialysis Therapy) [1], the target Hb levels were set at 11 to 12 g/dL (10-13 g/dL as a therapeutic window). The initial dose of Epoetin β was 1500 IU per HD session, 3 times a week (a total of 4,500 IU per week or 18,000 per 4 weeks). As for Darbepoetin α and Epoetin-β pegol, the corresponding doses were used as an initial one (10-40 μg once week and 40-160 μg every 4 weeks, respectively). As a principal, iron therapy was also performed according to the Guidelines for Renal Anemia in Chronic Kidney Disease)[1]. Iron (40 mg) was administrated intravenously a total of 13 times at every dialysis session with consideration of Hb levels. Iron status was re-evaluated a week or more after the final iron injection. Generally, iron therapy was initiated when patients showed TSAT levels below 20% or ferritin levels below 100 ng/mL in addition to Hb levels below 10 g/dL

despite appropriate ESA therapy. Therefore, patients with iron therapy (n=12) had a significantly higher level of ERI than those without iron therapy (n=155), while no significant difference in reticulocyte count was observed between patients with and without iron therapy.

1. Tsubakihara, Y. *et al.* 2008 Japanese Society for Dialysis Therapy: guidelines for renal anemia in chronic kidney disease. *Therapeutic apheresis and dialysis : official peer-reviewed journal of the International Society for Apheresis, the Japanese Society for Apheresis, the Japanese Society for Dialysis Therapy* **14**, 240-275, doi:10.1111/j.1744-9987.2010.00836.x (2010).

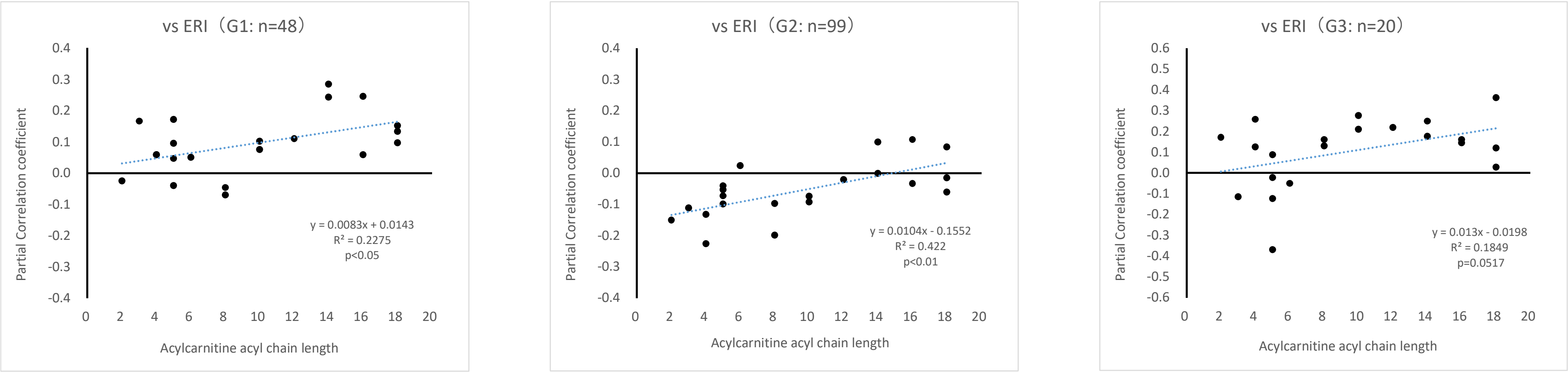

Supplementary Figure S1.
Scatter plots with a trend line between acylcarnitine chain length and partial correlation coefficients between each acylcarnitine and ERI in the G1, G2, and G3.
Partial correlation coefficients for ERI were adjusted for age, sex and log(HD vintage).

Supplementary Table S1. Univariate and stepwise multivariate regression analysis of variables affecting serum carnitine levels in the whole group

Variables	Univariate linear regression analysis			Stepwise multivariate linear regression analysis							
				Adjusted R^2 = 0.313			Bootstrap Results (2000 Replicas)				
	β	95% CI	P [a]	β	95% CI	P [a]	bias	SE	median	95% CI	P [a]
Sex (male=1, female=0)	0.243	0.094 to 0.392	**0.002**	–		0.970					
Age (year)	−0.295	−0.442 to −0.149	**<0.001**	−0.252	−0.395 to −0.109	**0.001**	0.0047	0.085	−0.237	−0.402 to −0.062	**0.001**
log (HD vintage, month)	−0.293	−0.440 to −0.146	**<0.001**	−0.257	−0.417 to −0.108	**0.001**	0.0086	0.090	−0.272	−0.445 to −0.089	**<0.001**
DM (yes=1, no=0)	−0.115	−0.269 to 0.038	0.140								
HT (yes=1, no=0)	0.074	−0.078 to 0.224	0.340								
Smoking (yes=1, no=0)	0.077	−0.076 to 0.231	0.321								
Dry weight (kg)	0.346	0.202 to 0.490	**<0.001**	–		0.836					
log (TG, mg/dL)	0.043	−0.111 to 0.197	0.581								
TC (mg/dL)	−0.064	−0.218 to 0.089	0.408								
HDL-C (mg/dL)	−0.037	−0.188 to 0.114	0.631								
LDL-C (mg/dL)	−0.115	−0.268 to 0.037	0.138								
Adiponectin (μg/mL)	−0.222	−0.372 to −0.072	**0.004**	–		0.201					
Fe (μg/dL)	0.144	−0.009 to 0.296	0.064								
TSAT (%)	0.094	−0.059 to 0.247	0.226								
Zn (μg/dL)	0.155	0.003 to 0.307	**0.046**	–		0.105					
RBC (10^4/μL)	0.080	−0.073 to 0.233	0.306								
Hemoglobin (g/dL)	−0.010	−0.163 to 0.144	0.903								
Reticulocyte counts (10^4/μL)	0.112	−0.041 to 0.264	0.151								
WBC (/μL)	0.159	0.007 to 0.310	**0.041**	–		0.251					
PLT (10^4/μL)	0.116	−0.036 to 0.269	0.134								
log (CRP, mg/dL)	0.075	−0.078 to 0.229	0.333								
log (Ferritin, ng/mL)	0.030	−0.124 to 0.184	0.701								
Albumin (g/dL)	0.182	0.030 to 0.333	**0.019**	–		0.449					
AST (U/L)	−0.060	−0.213 to 0.093	0.442								
ALT (U/L)	0.205	0.055 to 0.356	**0.008**	–		0.213					
ALP (U/L)	−0.010	−0.164 to 0.144	0.897								
LDH (U/L)	0.040	−0.114 to 0.193	0.609								
Kt/V	−0.328	−0.473 to −0.184	**<0.001**	−0.154	−0.303 to −0.003	**0.044**	−0.0027	0.077	−0.135	−0.289 to 0.014	**0.039**
PCR (g/day)	0.328	0.185 to 0.470	**<0.001**	0.236	0.097 to 0.379	**0.001**	0.0008	0.066	0.232	0.099 to 0.363	**0.001**
ERI [μg(4 weeks)/kg/(g/dL)]	−0.152	−0.304 to −0.00005	**0.050**	–		0.886					

a: P-values in bold are statistically significant.

Supplementary Table S2. Univariate and multivariate regression analysis using the forced entry method of variables affecting reticulocyte counts in the G1

Variables	Univariate linear regression analysis			Multivariate linear regression analysis using the forced entry method							
				Adjusted R^2 = 0.270			Bootstrap Results (2000 Replicas)				
	β	95% CI	P [a]	β	95% CI	P [a]	bias	SE	median	95% CI	P [a]
Sex (male=1, female=0)	−0.289	−0.573 to −0.005	**0.046**	−0.070	−0.362 to 0.221	0.628	0.002	0.111	−0.066	−0.295 to 0.137	0.528
Age (year)	0.023	−0.274 to 0.320	0.877								
log (HD vintage, month)	0.018	−0.279 to 0.315	0.904								
DM (yes=1, no=0)	−0.282	−0.567 to 0.002	0.052								
HT (yes=1, no=0)	−0.144	−0.412 to 0.146	0.327								
Smoking (yes=1, no=0)	−0.117	−0.412 to 0.177	0.427								
Dry weight (kg)	−0.110	−0.405 to 0.185	0.458								
log (TG, mg/dL)	0.287	0.003 to 0.572	**0.048**	0.183	−0.086 to 0.452	0.177	0.010	0.121	0.186	−0.016 to 0.450	0.158
TC (mg/dL)	0.011	−0.286 to 0.308	0.942								
HDL−C (mg/dL)	−0.054	−0.356 to 0.249	0.723								
LDL−C (mg/dL)	−0.064	−0.361 to 0.232	0.663								
Adiponectin (μg/mL)	0.031	−0.265 to 0.328	0.832								
Fe (μg/dL)	−0.235	−0.523 to 0.054	0.108								
TSAT (%)	−0.195	−0.486 to 0.096	0.183								
Zn (μg/dL)	0.054	−0.242 to 0.350	0.716								
RBC (10^4/μL)	0.071	−0.225 to 0.367	0.634								
Hemoglobin (g/dL)	0.215	−0.075 to 0.504	0.143								
WBC (/μL)	0.081	−0.215 to 0.377	0.584								
PLT (10^4/μL)	0.217	−0.072 to 0.507	0.138								
log (CRP, mg/dL)	−0.025	−0.322 to 0.271	0.864								
log (Ferritin, ng/mL)	0.047	−0.250 to 0.343	0.751								
Albumin (g/dL)	−0.149	−0.448 to 0.151	0.322								
AST (U/L)	−0.181	−0.473 to 0.111	0.218								
ALT (U/L)	−0.075	−0.371 to 0.221	0.612								
ALP (U/L)	0.132	−0.162 to 0.426	0.371								
LDH (U/L)	0.036	−0.260 to 0.333	0.806								
Kt/V	0.100	−0.195 to 0.396	0.498								
PCR (g/day)	−0.054	−0.357 to 0.249	0.721								
ERI [μg(4 weeks)/kg/(g/dL)]	0.164	−0.129 to 0.456	0.267								
iPTH (pg/mL)	−0.003	−0.305 to 0.300	0.986								
log (free carnitine, nmol/mL)	0.077	−0.219 to 0.373	0.602								
log (C2, nmol/mL)	0.383	0.108 to 0.657	**0.007**								
log (C16, nmol/mL)	0.363	0.086 to 0.639	**0.011**								
log (C18:1, nmol/mL)	0.480	0.220 to 0.740	**0.001**	0.410	0.120 to 0.701	**0.007**	0.003	0.159	0.396	0.152 to 0.771	**0.005**
log [C2/(C16+C18:1)]	−0.136	−0.405 to 0.154	0.355								

a: P−values in bold are statistically significant. Among acylcarnitines identified as a univariate significant factor, log (C18:1) was selected for multivariate analysis due to regression coefficient magnitude.
